# Supplementary material for: Pressure stabilizes ferrous iron in bridgmanite under hydrous deep lower mantle conditions
Source: Nat Commun. 2024 May 21;15:4333. doi: 10.1038/s41467-024-48665-8 (PMC11109188; doi:10.1038/s41467-024-48665-8)
Supplement: Supplementary file 1 — Supplementary Information [file 41467_2024_48665_MOESM1_ESM.pdf]

Table S1. Comparison of chemical composition of the recovered bridgmanite phase under hydrous versus dry deep lower mantle conditions. The chemical composition of the bridgmanite phase in each run were obtained using energy dispersive spectroscopy (EDS) in different transmission electron microscopes (TEM), JEM-ARM200F or FEI Talos F200X, and the chemical standards may slightly vary in different measurements. The numbers in parenthesis are one standard deviation based on multiple analyses, where X_{Fe} is iron content in atoms per two-cation formula unit. The bulk composition in the Run#344-102d was obtained with X_{Fe}=0.15 in the 5×5 μm^2 area of the heated center, an indication of no Fe loss compared to the starting material Fs15.

Run#		O (at.%)	Mg (at.%)	Si (at.%)	Fe (at.%)	X_{Fe}
390-93	Bridgmanite / hydrous			105 GPa&2250 K		
	#1	60.30	17.64	19.53	2.53	
	#2	59.60	17.54	20.08	2.77	
	#3	60.27	17.29	20.05	2.39	
	#4	59.96	17.35	19.66	3.04	
	#5	60.66	17.02	19.35	2.97	
	Avg	60.16(0.14)	17.37(0.27)	19.73(0.20)	2.74(0.21)	0.14(1)
344-102d-2	Bridgmanite / dry			115 GPa&2350 K		
	#1	61.35	19.53	18.73	0.39	
	#2	61.57	19.53	18.64	0.26	
	#3	59.54	20.18	19.94	0.34	
	#4	61.36	19.93	18.46	0.26	
	Avg	60.96(0.82)	19.79(0.28)	18.94(0.58)	0.31(0.06)	0.02(0.3)
344-102d	Bridgmanite / dry			114 GPa&2200 K		
	#1	59.33	18.39	20.07	2.21	
	#2	59.18	18.26	20.35	2.21	
	#3	59.08	18.13	20.28	2.51	
	#4	58.66	18.92	20.38	2.05	
	Avg	59.06(0.25)	18.43(0.30)	20.27(0.12)	2.25(0.17)	0.11(1)
	Bulk	58.66	17.49	20.87	2.98	0.15
		O (at.%)	Mg (at.%)	Si (at.%)	Fe (at.%)	Al (at.%)
344-108m	Al^{3+}-bearing bridgmanite / hydrous			120 GPa&2200 K		
	#1	59.48	12.3	18.66	6.18	3.38
	#2	58.32	12.4	18.87	6.12	4.29
	#3	59.94	11.03	17.87	6.70	4.46
	Avg	59.25(0.68)	11.91(0.62)	18.47(0.43)	6.33(0.26)	4.04(0.47)

Fig. S1 Ferric iron concentration in dry aluminous bridgmanite from previous studies. The symbols represent the data for aluminous bridgmanite [1-6]. The red dash square includes the data points over the pressure range of 80-110 GPa. Note that all the pressures were recorded after the temperature quench. For all the data included in the red square, the Al^{3+} content in bridgmanite was less than 0.1 per two-cation formula unit but the ferric iron ratios are scattered.

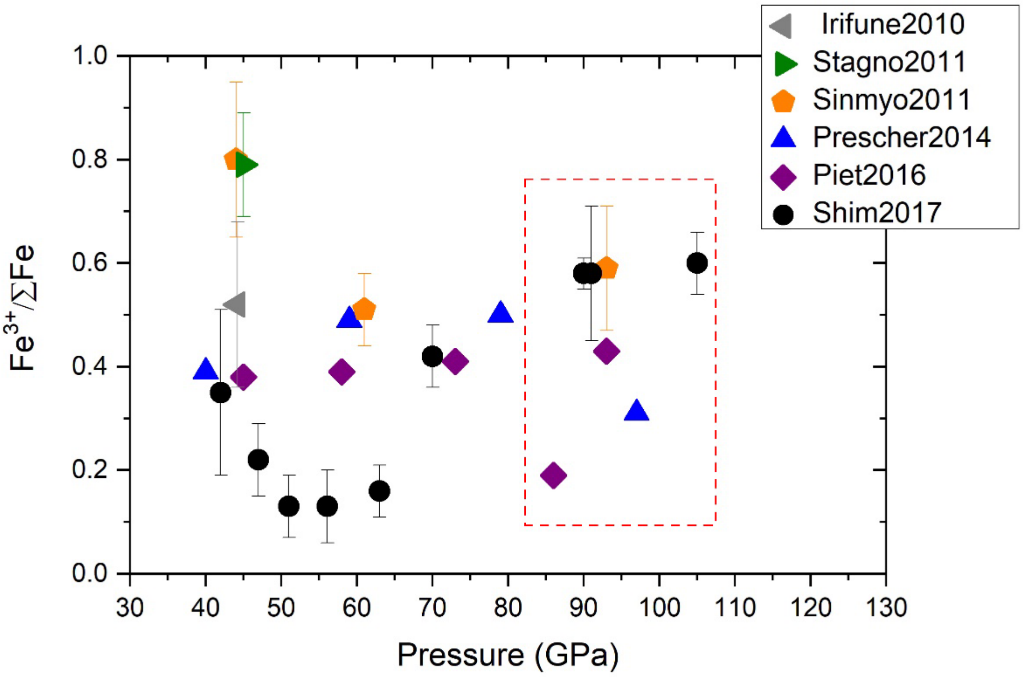

Fig. S2 Chemical analysis of the recovered sample at 125 GPa and 2350 K (Run#390-112). (A) A thin section showing a clear boundary between the hydrous SiO_2 layer and bridgmanite $(Mg,Fe)SiO_3$. (B) High-angle annular dark-field (HAADF) scanning TEM image of a selected region and (C-F) element mapping of the region suggests a pure bridgmanite phase. No detectable iron metal was observed.

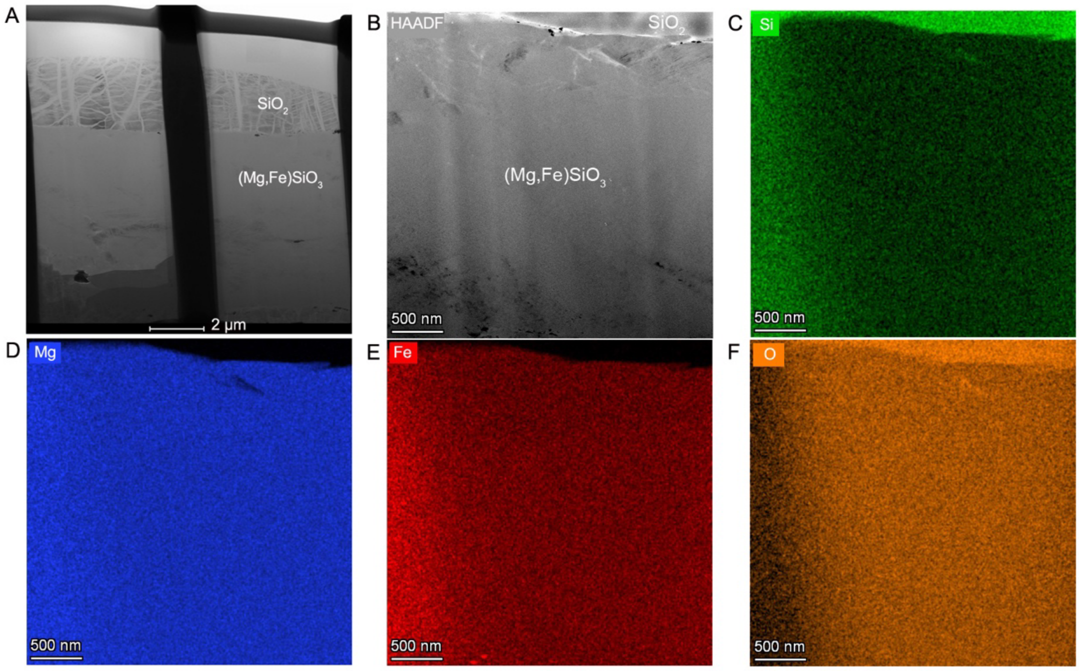

Fig. S3 Transmission electron microscope (TEM) images of the recovered sample showing the coexistence of Fe-depleted bridgmanite, a mixture of Fe-rich grains (bright particles) and a silica-rich amorphous phase (Run#344-102d, 114 GPa&2200 K). High-angle annular dark-field (HAADF) scanning TEM images showing (A) a section over ~15 μm length across more than half of the heated spot along the radial direction and (B) a selected area in the heated center as indicated by the rectangle box in (A). The chemical analysis listed in Table S1 indicates Fe-depletion in bridgmanite with X_{Fe}=0.11(1) while the bulk composition of the whole area in (B) remained identical to the starting material with X_{Fe}=0.15.

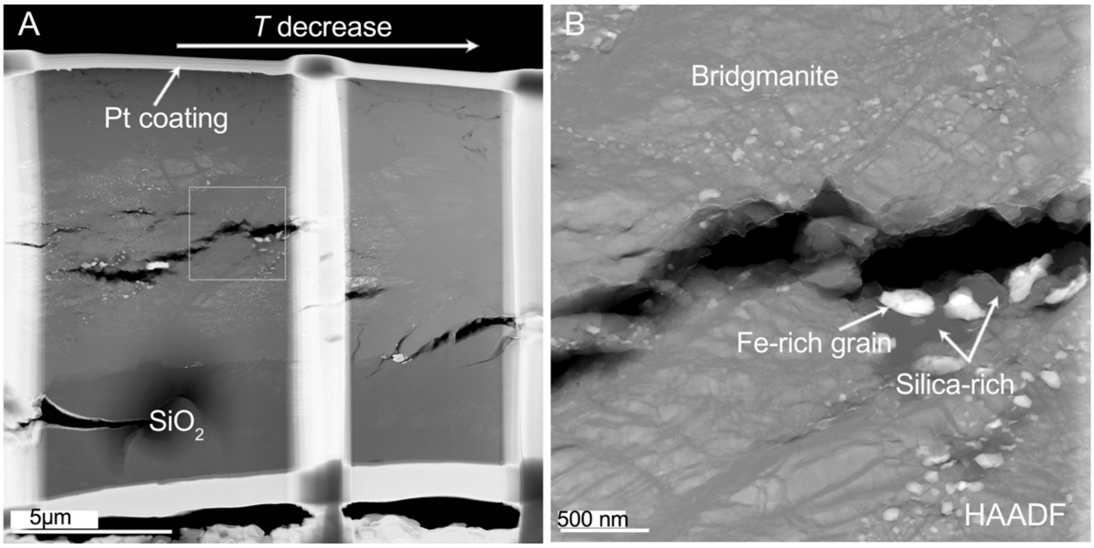

Fig. S4 Representative XRD images and patterns of the samples collected at high pressure and temperature quench. (A) 112 GPa and after temperature quench from 2400 K (Run#344-112) versus (B) 81 GPa and after temperature quench from 1950 K (Run#275-81). (C) The XRD pattern collected at 93 GPa and after *T* quench from ~2250 K (Run#390-93) and (D) collected at 112 GPa and after *T* quench from ~2400 K (Run#344-112), respectively, showing the coexistence of bridgmanite (Brd) and NiAs-type silica (Nt) phases. The X-ray wavelength was 0.6199 Å.

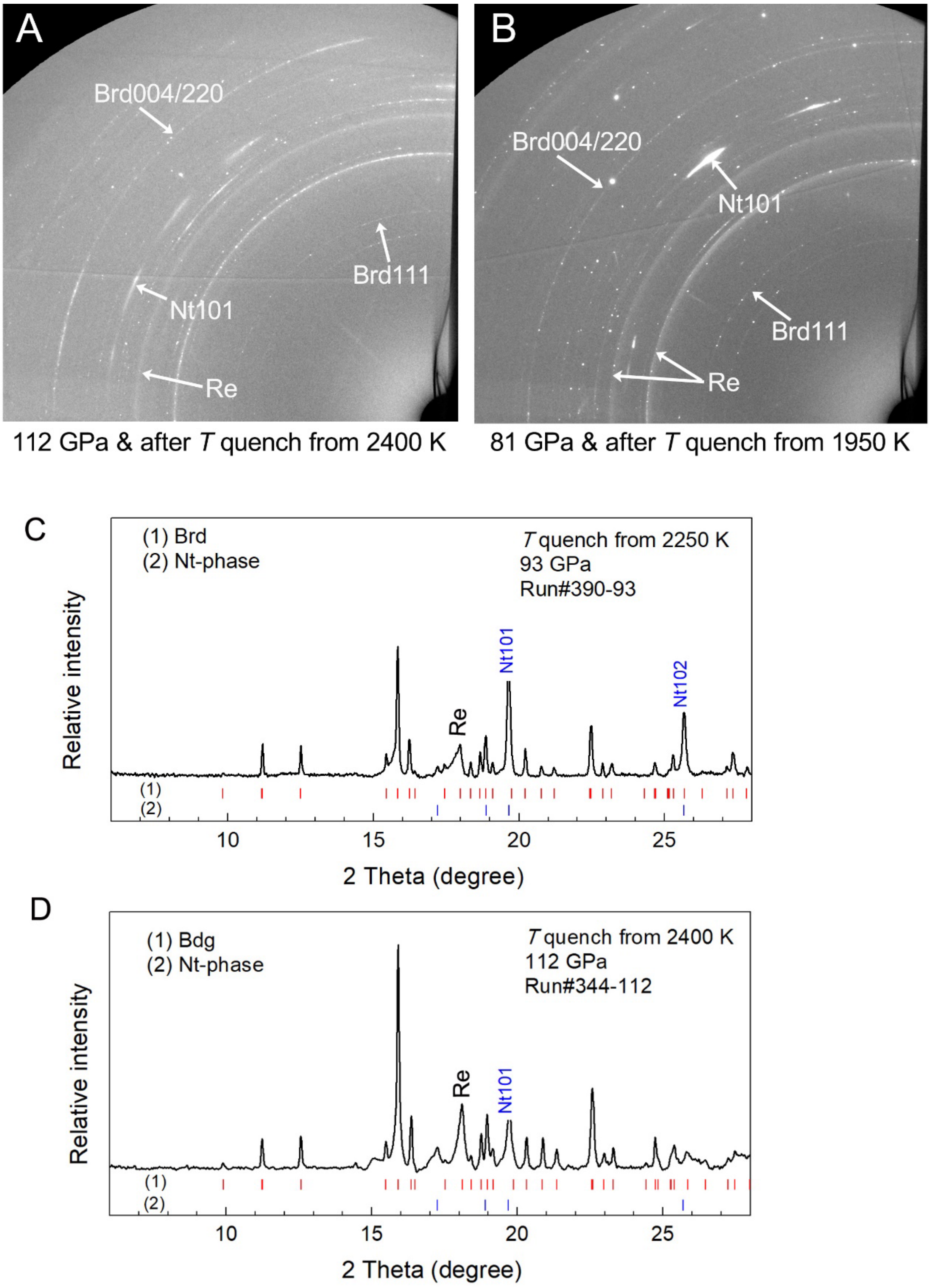

Fig. S5 Determination of the Fe^{3+}/∑Fe ratio by fitting Fe L_3 reference spectra of the ferrous ($FeCO_3$) and ferric iron (Fe_2O_3) standards. EELS data of (A) bridgmanite (Run#390-93) and (B) Fs15 opx starting material are presented. The red and blue curves are the measurements of Fe^{2+} and Fe^{3+} standards, respectively. The black dots are the measurements of the samples and the green curve is the calculated spectrum fitted by a linear combination of Fe L_3 reference spectra of the Fe^{2+} and Fe^{3+} standards from 703 to 717 eV. The black thin line represents the residual of the fit.

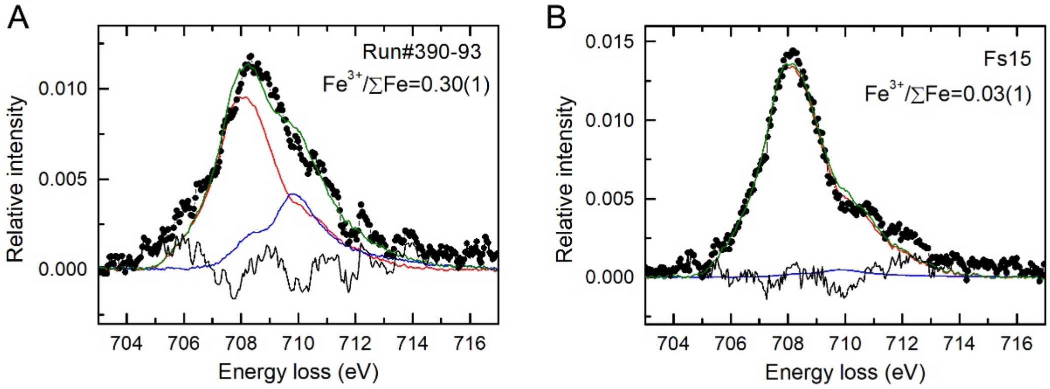

Fig. S6 Experiment conducted at 91 GPa and 1950 K with Fs15 sandwiched between hydrous silica gel (Run#275-81). (A) The XRD pattern was collected at 81 GPa and room temperature after temperature quench from ~1950 K and (B) the TEM image on the recovered sample showed the coexistence of bridgmanite, Fe metal (bright small grains) and a silica phase. (C) The EELS data of the amorphous bridgmanite indicated pure Fe^{3+}, in comparison with the data of Fs15 starting material and Fe_2O_3 ($Fe^{3+}/\Sigma Fe$= 100%). The collection of EELS was carried out in the area free of Fe metal as shown by the circle in (B).

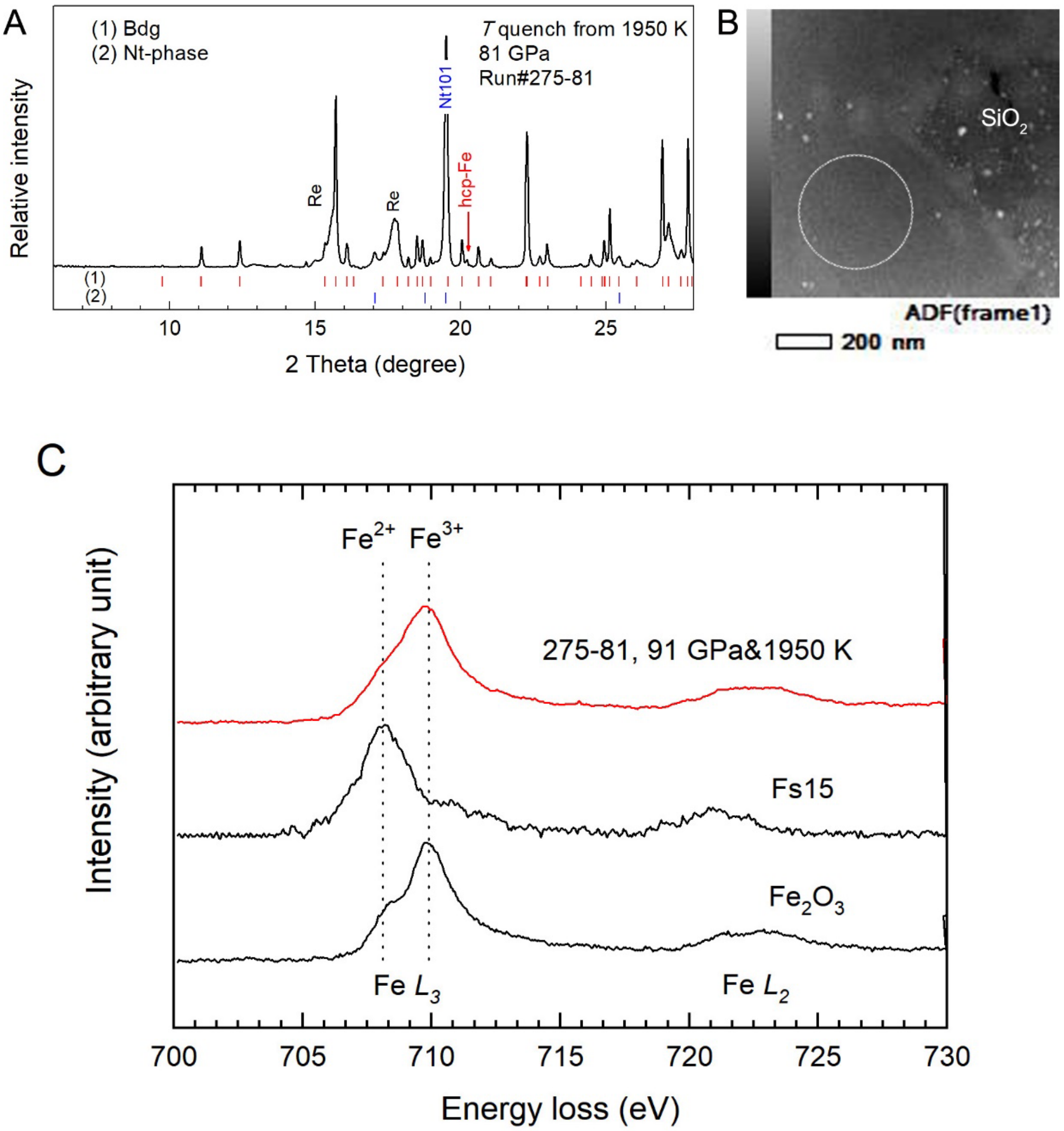

Fig. S7 Recovered sample from 92 GPa and 1850 K (Run#332-82). The TEM image (A) shows the coexistence of bridgmanite and Fe metal and the SAED data (B) indicate the crystalline nature of the recovered bridgmanite. The element mapping of Mg (C) and Fe (D) shows the distribution of Fe metal particles in the sample. The EELS data (E) suggested a mixture of Fe^{2+} and Fe^{3+} with an average $Fe^{3+}/\sum Fe$ of 0.56(6). For this dataset, two integration windows of 2 eV width were applied to the Fe $L_{2,3}$ edges from 708.5 eV to 710.5 eV and from 719.7 eV to 721.7 eV, respectively, and the $Fe^{3+}/\sum Fe$ ratio was obtained by applying the ratios of integrated intensity to the universal curve determined in a previous study[7]. The maxima of Fe^{2+} (707.8 eV) and Fe^{3+} (709.5 eV) at the L_3-edge are indicated by the black dotted lines.

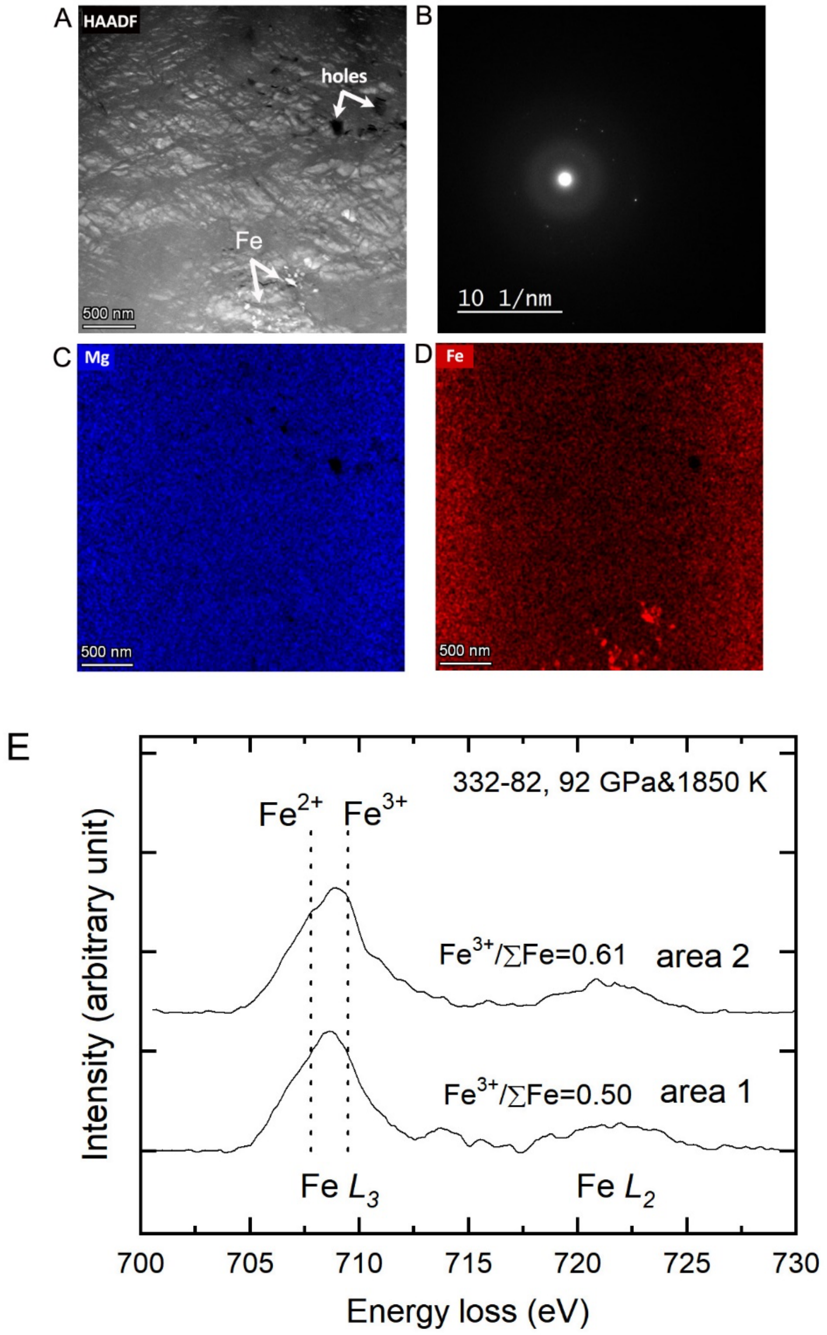

Fig. S8 X-ray diffraction pattern of Fs15 composition sandwiched between dry silica layers collected at 99 GPa and after temperature quench from 2350 K (Run#344-99d). (A) A selected area of the two-dimensional diffraction pattern and (B) the integrated diffraction pattern showing weak peaks of the H-phase in coexistence with bridgmanite. Broad peaks of the NiAs-type (Nt) structured silica indicate that the silica pressure medium was converted to the Nt-phase in a metastable state at similar conditions for the observation of Nt-phase in dynamic compression[8]. We obtained the unit-cell parameters for the bridgmanite (Brd) phase with a=4.3568(7) Å, b = 4.5913(15) Å, c = 6.3373(15) Å and V = 126.77(6) Å^3 at 99 GPa. The X-ray wavelength was 0.2900 Å.

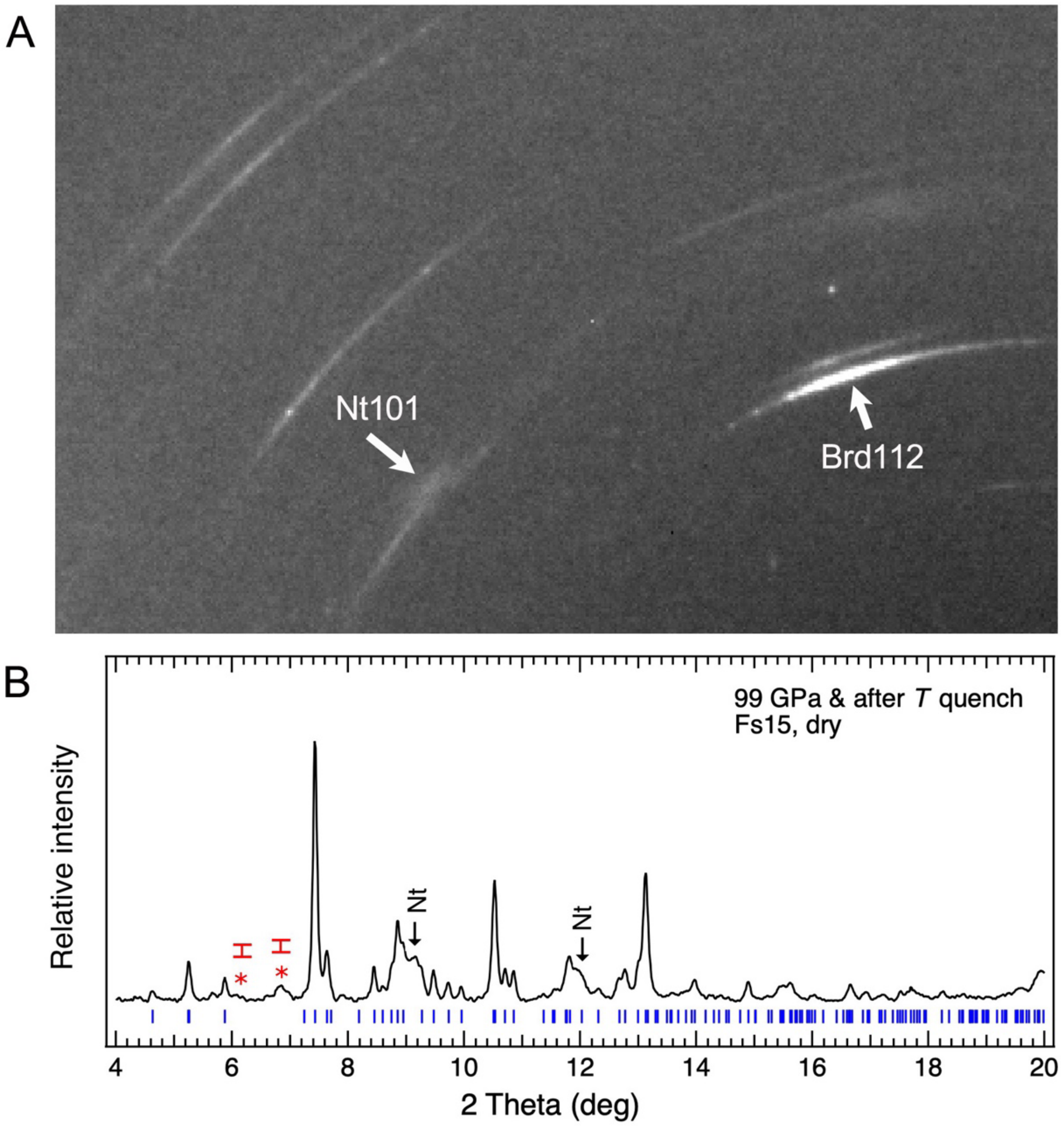

Fig. S9 TEM image and EELS data of the aluminous bridgmanite phase recovered from 120 GPa and 2200 K under hydrous conditions. We used a gel starting material with composition of 24.9 mol% MgO-12.8 mol% Al_2O_3-7.5 mol% Fe_2O_3-54.8 mol% SiO_2 containing ~4 wt% H_2O (Run#344-108m). (A) Coexistence of Al^{3+}-bearing bridgmanite (Brd) with NiAs-type silica phase (Nt) phases. We conducted two consecutive EELS measurements on one selected bridgmanite grain and collected SAED data before and after each EELS measurement. We observed gradual amorphization of bridgmanite during the measurement, which led to an increase of the $Fe^{3+}/\sum Fe$ ratio from 0.46 (#1) to 0.75 (#2) as shown in (B).

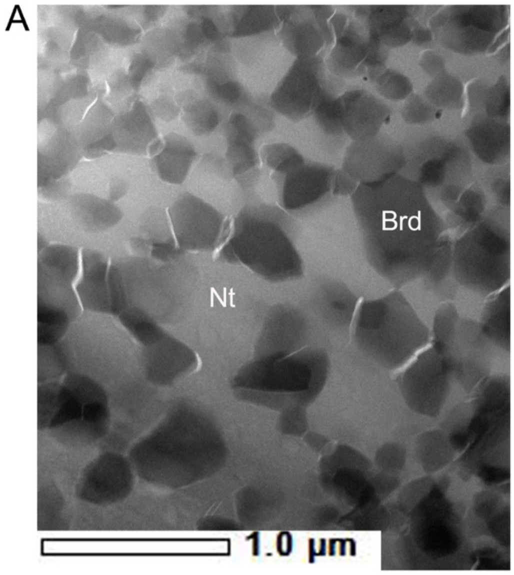

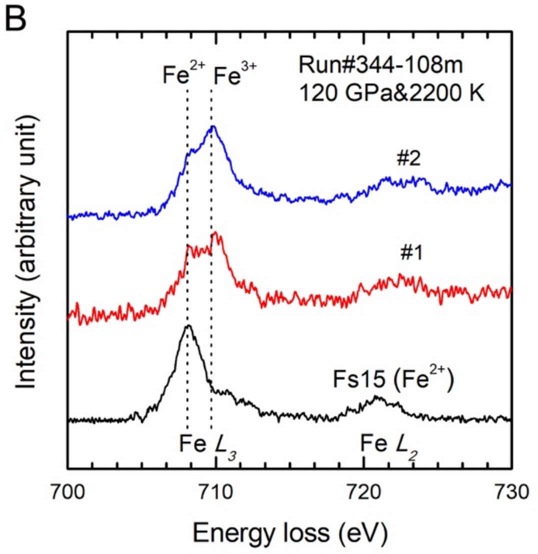

Supplementary Note 1. In an experiment conducted at 112 GPa and 2350 K (Run#344-99d), we performed XRD measurements at 99 GPa after temperature quench. Only weak diffraction peaks of the H-phase were observed while the texturing in the diffraction pattern of bridgmanite (**Fig. S8**) indicates a nanocrystalline sample with lattice preferred orientation possibly due to non-hydrostaticity in a solid pressure medium. No diffraction for any Fe metal or Fe oxides was observed. Weak diffraction of the H-phase, in contrast to a large proportion of Fe-rich component in coexistence with Fe-depleted bridgmanite in the recovered sample, implies poor crystallization of the H-phase in a solid pressure medium. In the previous study[9], a thin opx sample was loaded in Ne pressure medium and sharp diffraction peaks of the H-phase were observed.

Supplementary References:

1. Irifune T, Shinmei T, McCammon CA, Miyajima N, Rubie DC, Frost DJ. Iron Partitioning and Density Changes of Pyrolite in Earth's Lower Mantle. *Science* **327**, 193-195 (2010).
2. Sinmyo R, Hirose K, Muto S, Ohishi Y, Yasuhara A. The valence state and partitioning of iron in the Earth's lowermost mantle. *Journal of Geophysical Research: Solid Earth* **116**, B07205 (2011).
3. Stagno V, Tange Y, Miyajima N, McCammon CA, Irifune T, Frost DJ. The stability of magnesite in the transition zone and the lower mantle as function of oxygen fugacity. *Geophysical Research Letters* **38**, L19309 (2011).
4. Prescher C, Langenhorst F, Dubrovinsky LS, Prakapenka VB, Miyajima N. The effect of Fe spin crossovers on its partitioning behavior and oxidation state in a pyrolitic Earth's lower mantle system. *Earth and Planetary Science Letters* **399**, 86-91 (2014).
5. Piet H*, et al.* Spin and valence dependence of iron partitioning in Earth's deep mantle. *Proceedings of the National Academy of Sciences* **113**, 11127-11130 (2016).
6. Shim S-H*, et al.* Stability of ferrous-iron-rich bridgmanite under reducing midmantle conditions. *Proceedings of the National Academy of Sciences* **114**, 6468-6473 (2017).
7. van Aken PA, Liebscher B. Quantification of ferrous/ferric ratios in minerals: new evaluation schemes of Fe L23electron energy-loss near-edge spectra. *Physics and Chemistry of Minerals* **29**, 188-200 (2002).
8. Tracy SJ, Turneaure SJ, Duffy TS. Structural response of α-quartz under plate-impact shock compression. *Science Advances* **6**, eabb3913 (2020).
9. Zhang L*, et al.* Disproportionation of (Mg,Fe)SiO3 perovskite in Earth's deep lower mantle. *Science* **344**, 877-882 (2014).
